# How the £100,000 Is to Be Distributed

**Published:** 1919-12-06

**Authors:** 


### THE RED CROSS GRANT TO METROPOLITAN HOSPITALS.

### How the £100,000 is to be Distributed.

King Edward's Hospital Fund for London has issued a preliminary notice as to how the distribution of £100,000, out of the surplus funds of the Joint Committee of the British Red Cross Society and the Order of St. John of Jerusalem, is to be carried out.

This large sum will be dealt with entirely separate from the ordinary distribution of the King's Fund, and will take place early in 1920, but the Fund desires no applications to be sent in until a further notice has been issued, probably in January 1920.

The distribution will not extend to institutions which do not possess the constitution and characteristics of a voluntary hospital, or those which have no beds for in-patients or for the treatment of incurable or mental cases, or are not situated in the County of London or within nine miles of Charing Cross.

Amongst the conditions attaching to the special distribution will be the following:—

A hospital will not be eligible to participate unless it can and will treat ex-sailors or ex-soldiers. This condition excludes, for instance, hospitals which treat only women and children.

Grants will be made only towards definite schemes for extension and improvements. Nothing will be given for endowment or maintenance or for the payment of debts. All schemes must first be submitted and passed by the King's Fund, in accordance with the usual procedure.

No grant will be paid until the Fund is satisfied that the scheme is matured and that either the balance required for it, or a sum equal to the grant (whichever is less) has been raised. If these conditions remain unfulfilled on March 31, 1920, the grant will lapse, unless otherwise ordered. The Fund will require a definite undertaking in writing that the grant will be used only for the purpose for which it is given.

A hospital receiving a grant of £1,000 or over, which does not already publish its accounts in accordance with the revised uniform system, will be required to come into line with other hospitals in this respect, beginning with the accounts of the year 1921.

The above is the substance of the circular of King Edward's Hospital Fund for London. It is possible that there may be some condition attached to the gift of the surplus funds of the Joint Committee, which has forced the hands of the Fund, in respect of the important decision that the whole of the large sums to be allocated must be spent on structural improvements and alterations, and, presumably, necessary equipment, and further that such works must be carried out within one year of the grant. This period will cover a time when a large number of those engaged in the building trade will be absorbed in the labour required for the construction of houses, and the conversion of houses into flats, under the national housing movement. Labour of this kind will be dear and scarce, and it is probable that money spent by hospitals in this way would go a great deal further in a few years time. Further, the acquisition of necessary sites will take place at a very unfavourable time. Money for extensions could hardly secure smaller value later than it does now, when a large sum spent on alterations or improvements to a building produces a result which is often ludicrously small.

Why is the policy of the King's Fund (and possibly that of the Joint Committee) expressed in this way? It can hardly be because more beds are required for the discharged sailor and soldier, whose needs are rapidly becoming a question of out-patient rather than in-patient treatment. Is it in order to secure extension to meet the notorious difficulty of the inflated waiting-list, using such an extension for a brief time for ex-Service men, technically to meet the objection that Red Cross funds must be used in a Red Cross manner?

None have recognised the gravity of the position as regards the waiting-list evil more clearly than The Hospital. It is a question which must be tackled from within or from without the hospitals at the earliest possible moment. But many will regret to see an important sum largely thrown away at a moment when labour has so poor a perception of the needs of the national position; one when ten bricks are laid in a time which fully suffices for the laying of forty, and when a surface yard of paintwork is covered while twice the area could have been dealt with.

Then it is quite possible that in the short time before applications are to be made that hospitals will carry their spirit of competition to the extent of putting forward schemes which are but half formulated and of doubtful necessity.

To many it would appear wiser that the King's Fund should, with the consent of the Joint Committee of the British Red Cross Society and the order of St. John of Jerusalem modify the conditions. Why should not the £100,000 be kept in hand to be used for capital purposes when better value can be obtained, and when schemes for enlargement are more matured than they would be after the hasty conception and development possible in a few weeks, broken by a holiday period and the inevitable administrative pressure that always occurs at the end of a financial year?

